# S11-3 Implementation of Community-Based Physical Activity Promotion Focusing on Individuals with Social Disadvantages, Developed Using a Participatory Approach

**DOI:** 10.1093/eurpub/ckac093.057

**Published:** 2022-08-29

**Authors:** Jana Semrau

**Affiliations:** Department of Sport Science and Sport, FAU Erlangen-Nürnberg, Erlangen, Germany

**Keywords:** cooperative planning, social disadvantages, community-based physical activity, guidelines, co-creation

## Abstract

**Background:**

Recently, the German Federal Ministry of Health has provided funding aimed at the sustainable implementation of the National Recommendations for Physical Activity and Physical Activity Promotion in German municipalities. This presentation reports on an ongoing project that employs a participatory action model for population-based physical activity (PA) promotion in these municipalities and gives insights into real-world experiences made during project implementation.

**Methods:**

Using a participatory approach, an action model for PA promotion was developed in collaboration with 64 nationwide stakeholders (Phase I). Based on systematic criteria and in cooperation with the Federal Centre for Health Education (BzGA) and the German Statutory Health Insurance (GKV), three rural districts and three cities were then selected as pilot municipalities. In phase II, the action model was implemented in these six communities. Implementation steps included (1) preparation, (2) assessment, and (3) setting up a steering committee and a cooperative planning group in all communities. The entire process was closely coordinated with local administrations. Currently, cocreated measures adapted to local needs are being (4) developed and (5) implemented, with a particular focus on improving access to the development and usage of these measures for people with social disadvantages.

**Results:**

Preliminary results indicate that steps (1) to (3) have been successfully implemented in all pilot municipalities. ‘Champions’ (persons committed to the local project) were found to be particularly important for each step. The involvement of political stakeholders (n = 2-13), experts (n = 2-12), representatives from the administration (n = 8-25), multipliers (n = 5-16), and citizens (n = 0-4) in the cooperative planning process varied across pilot municipalities. Participants from the administration represented different sectors (e.g. city planning, sports, health administration). Preliminary results show that a broad range of measures was developed to promote personal skills, infrastructures, policy actions, and community action for PA.

**Conclusions:**

The action model proved suitable to promote PA in the pilot municipalities. In all six cases, adaptations had to be made to accommodate the intervention to the respective community-specific context. Our results indicate that involving individuals from a socially disadvantaged background remains a particular challenge of participatory PA promotion.

